# Quality indicators in living kidney donation for transplantation: a scoping review

**DOI:** 10.3389/fmed.2026.1698289

**Published:** 2026-05-08

**Authors:** Rafael Rodrigo Da Silva Pimentel, Luiza Ribeiro Cezar, Nathalia Izilda Xavier De Alcantara, Carolina Miranda Muniz, Íris Jardim Souza, Maristela Santini Martins, Marcelo José Dos Santos

**Affiliations:** 1 Center for Studies, Research, and Practice in Primary Health Care and Networks (CEPPAR), Hospital Israelita Albert Einstein, São Paulo, Brazil; 2 Professional Guidance Department, School of Nursing, University of São Paulo, São Paulo, Brazil; 3 JBI Brazilian Centre for Evidence-Based Health Care, São Paulo, Brazil

**Keywords:** kidney, kidney transplantation, living donors, quality indicators in health care, transplant (MeSH terms), health services administration, health services research

## Abstract

**Introduction:**

Living kidney donation has increased worldwide due to the growing demand for transplants. This demand has created a need to measure the quality of this service using quality indicators. Recognizing the importance of these indicators, this review aimed to identify and categorize quality indicators in living kidney donation for transplantation.

**Method:**

This scoping review was prepared according to the JBI method, previously registered in the Open Science Framework. The search was carried out between March and May 2023 in the following databases and libraries: Virtual Health Library, CINAHL, Embase, MEDLINE, Web of Science, together with gray literature platforms, such as Google Scholar, OpenGrey (GreyNet), CAPES Theses and Dissertations, Global ETD Search (previously: Union Catalog), Open Access Theses and Dissertations. Additionally, 17 official websites from donation, transplantation, and quality institutions were consulted. The guiding question was: what quality indicators are used in living kidney donation? Data selection and extraction were performed in pairs with consensus rounds. Included were documents published in any language since 1980, the year solid organ transplantation was established as the most successful treatment, along with policies and practices to improve donation and transplantation. A descriptive analysis was conducted to present the results.

**Results:**

The literature search identified 3,095 records, of which 14 met the inclusion criteria. These studies reported 89 metric mentions, consolidated into 74 unified quality indicators. The indicators varied widely in definition and classification and were distributed across multiple stages of the living kidney donation process, with a substantial concentration in the living-donation phase.

**Conclusion:**

The analysis revealed significant variations in the nomenclature and definitions of indicators as well as the unavailability of more detailed information.

## Introduction

Living kidney donation has become a globally recognized strategy for increasing organ supply and reducing waiting times. Its growing adoption is driven by two major factors: the continuous rise in demand for transplants and the pursuit of improved clinical outcomes (1). Despite these efforts, the number of donated organs meets only about 10% of the global need (2). In Brazil, for example, only 50% of the estimated requirement for kidney transplants was met in 2023, with 15% originating from living donors (3). These data underscore the importance of expanding living kidney donation as a means of mitigating organ shortages and improving access to transplantation.

The assessment of quality in healthcare is grounded in Donabedian’s conceptual framework, which defines quality as a combination of professional excellence, efficient use of resources, minimal risk, and high levels of patient and user satisfaction (4). Donabedian emphasized that quality can only be evaluated after establishing a clear definition of what the concept entails (4, 5). Quality management extends beyond service delivery itself to include the processes through which care is provided (6), and its multidimensional nature presents challenges for measurement (7). Establishing a shared understanding of the quality construct is therefore essential before attempting to assess or improve it (8, 9).

Among the several approaches to classifying quality indicators, Donabedian’s evaluative triad remains one of the most widely used frameworks. It comprises three components: structure, process, and outcomes (4, 5, 10). Structure refers to the physical, human, and organizational resources available for care; process encompasses the activities involved in delivering care, from diagnosis to patient education; and outcomes represent the changes in health status attributable to the care provided (11–13).

In addition, Donabedian also proposed seven pillars of quality. The first pillar, efficacy, refers to the institution’s ability to achieve desired results under optimal conditions. Efficiency relates to the appropriate use of resources to obtain the best possible outcomes at the lowest feasible cost. Effectiveness concerns the delivery of high-quality services in a timely manner to ensure positive results. Optimality focuses on eliminating unnecessary or inefficient processes. Acceptability emphasizes the provision of care that respects the autonomy, expectations, and values of patients and their families, ensuring a patient-centered experience. Legitimacy reflects how care is perceived by society, while equity involves providing care fairly, without discrimination based on beliefs, socioeconomic status, or other social factors (11–13).

In light of this conceptual complexity proposed by Donabedian, scoping reviews are particularly appropriate for examining challenging and multifaceted fields of research (14–16). This type of review follows a rigorous methodological process aimed at identifying existing gaps in the body of knowledge and supporting the planning of future investigations and policy development. Preliminary searches in PROSPERO, MEDLINE, the Cochrane Database of Systematic Reviews, and JBI Evidence Synthesis found no reviews specifically focused on quality indicators in living kidney donation, reinforcing both the relevance and the necessity of this study.

These considerations underscore the importance of evaluating the quality of care provided in living kidney donation. Accordingly, this study aimed to identify and categorize quality indicators used in living kidney donation for transplantation.

## Materials and methods

This scoping review was prepared according to the JBI (14) method and reported in accordance with the Preferred Reporting Items for Systematic Reviews and Meta-Analyses for Scoping Reviews (PRISMA-ScR) (17) extension, which was previously registered in the Open Science Framework (OSF) (18).

### Identifying the research question

This study’s research question was formulated according to the mnemonic combination PCC (14) (P: Population – living kidney donors; C: Concept – quality indicators; C: Context – Health services and systems), establishing the following guiding question:

- What are the quality indicators used in living kidney donation for transplantation?

### Eligibility criteria

The articles were refined based on pre-established eligibility criteria: studies presenting indicators to assess the quality of living kidney donation; primary and secondary research using any method; and white and gray literature in any language since 1980. From that year onward, solid organ transplantation was established as a successful treatment with improved donation and transplantation policies and practices (19). Conference proceedings, full texts unavailable in electronic media or after two contacts with the authors, websites/electronic portals with restricted access, books, book chapters and research projects were excluded.

### Search strategy

With the support of a librarian who is an expert in conducting reviews and a Core Staff member of JBI Brazil, the researchers developed the search strategy and executed it from March 9 to May 25, 2023. The terms “Quality Indicators,” “Quality Metrics,” “Health Status Indicators”, “Quality Indicators”, “Health Care”, “Kidney Transplantation”, “Living Donors”, “Living Donor Kidney” And “Kidney Donation in Life” and their variations were used, being adapted according to each database, portal or directory. The Boolean operators “AND” and “OR” were used. A detailed description of the search strategy can be found in Supplementary material 1.

The searches were conducted in the following databases and libraries: MEDLINE (NCBI); Scopus (Elsevier); Embase (Elsevier); Web of Science (Clarivate Analytics); CINAHL Complete (EBSCO); and Virtual Health Library (VHL) (Bireme). In gray literature: Google Scholar; OpenGrey (GreyNet); CAPES Theses and Dissertations; Global ETD Search (formerly Union Catalog); Open Access Theses and Dissertations. And in the following official websites: Transplantation Society (TTS); Global Observatory on Donation and Transplantation (GODT); *Organización Nacional de Trasplantes* (ONT); *Sociedad de Trasplante de América Latina y el Caribe* (STALYC); European Society for Organ Transplantation (ESOT); Healthcare Quality Improvement Partnership; National Quality Forum; International Society for Quality in Health Care (ISQua); Agency for Healthcare Research and Quality (AHRQ); National Institute for Health and Care Excellence (NICE); the Ministry of Health of Brazil; *Agência Nacional de Vigilância Sanitária* (ANVISA); and *Centro Colaborador para a Qualidade do Cuidado e Segurança do Paciente* (PROQUALIS).

### Screening of records

Initially, the records identified in the searches were condensed using the reference management software Zotero v. 5.0.95.1 (George Mason University, VA, United States) to eliminate duplicates. Two stages of reading—title, abstract, and full text—were developed in a Microsoft Excel 2016 spreadsheet, which included study identification data and a record of decisions made in each round. Pairs of reviewers performed exploratory reading of titles and abstracts independently, judging studies related to the research question that met the inclusion criteria. Disagreements were resolved through consensus meetings between peers or by assessment of a third reviewer, when necessary. The full texts of the pre-selected studies were read in full to assess the content and compliance with the objective/question and subsequent data synthesis. Subsequently, the reference lists of the included articles were checked for additional studies. All searches, decisions and steps were documented. The PRISMA-ScR flow diagram presented the screening of the included studies (17).

### Extracting data

During the data extraction stage of the studies included in the review, a Microsoft Excel 2016 instrument based on the JBI model was used. The instrument included the following variables: database; author(s); title; DOI/access link; year of publication; country; collection period; study location/institution; objectives; methods (study design, age/age range, sample size, data analysis, indicator validity, and indicator development project); and results (number of indicators, application context, and indicator file). A pilot study was conducted on some of the included records to adjust the data collection tool. Data extraction was performed in pairs with group consensus rounds.

### Summarizing and reporting results

A descriptive analysis was performed using the simple (*n*) and relative (%) frequencies of the findings in order to present the main characteristics of articles and indicators. After completing the analysis, the indicator files were organized, and duplicate indicators were consolidated. The results were categorized based on Avedis Donabedian’s framework (structure, process and outcomes) (10) and on the pillars of quality (efficiency, efficacy, effectiveness, optimality, acceptability, legitimacy, equity, security, centrality in care) also proposed by Avedis Donabedian and expanded by the Institute of Medicine (5, 10). Supplementary material 1 contains a document with a summary of all indicators.

All steps were conducted in pairs and discussed in consensus rounds with the review group. To communicate the results, a synoptic table was prepared with the main characteristics of studies aiming to present an overview of all the material.

## Result

**Figure 1:**
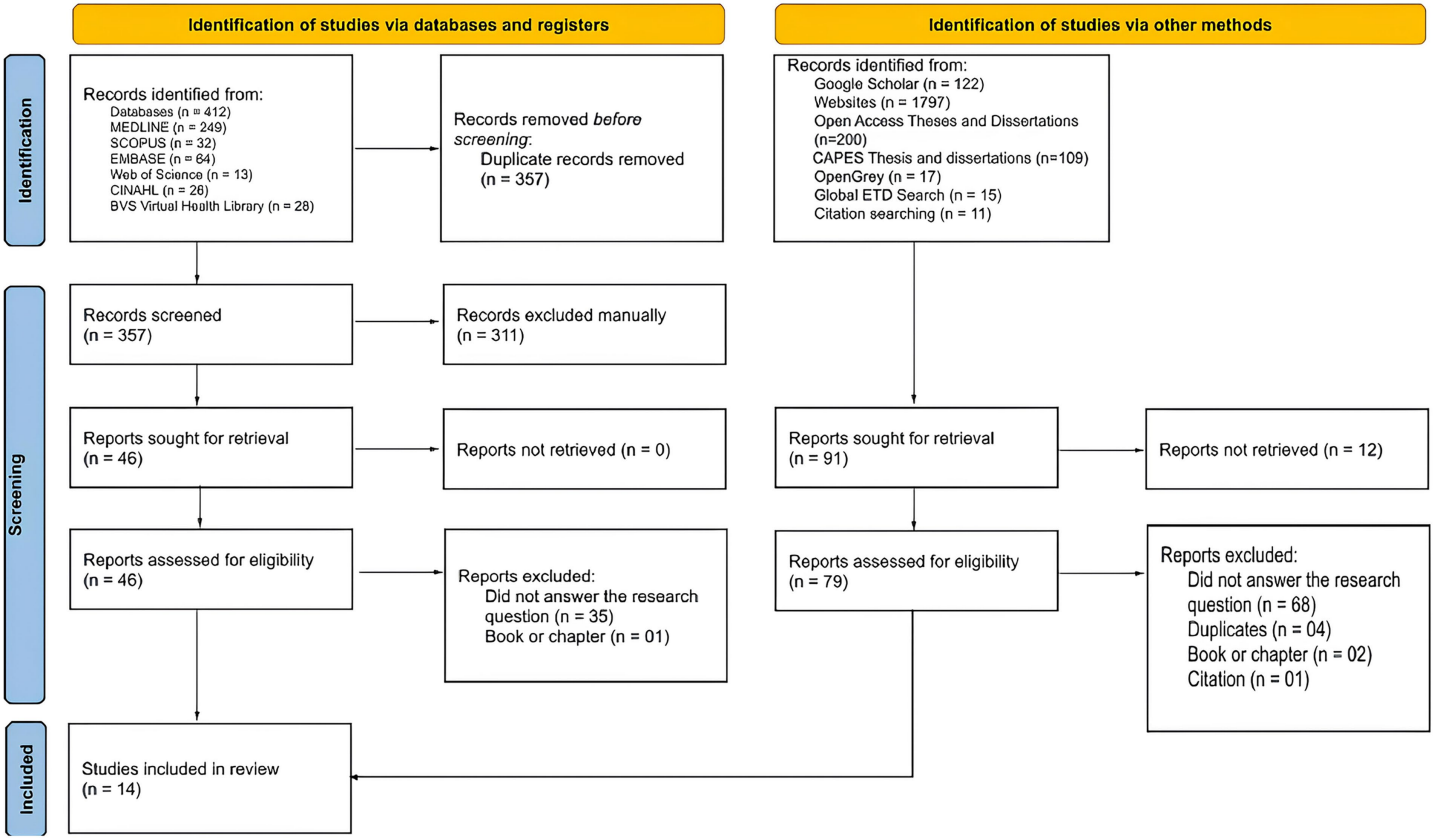
Flow diagram of study selection following the Preferred Reporting Items for Systematic Reviews and Meta-Analyses (PRISMA) model (São Paulo, SP, Brazil, 2026).

The literature search identified 3,095 records. After excluding duplicates, 2,738 titles and abstracts were reviewed, 125 full texts were analyzed, and 14 documents were included. The PRISMA flow diagram details the screening of records (Figure 1).

Of the included documents, most were original articles (*n* = 11, 78.58%), followed by manuals or guides (*n* = 2, 14.28%). Concerning characteristics of type, nature, and approach, half of articles were quantitative (*n* = 7, 50%), followed by methodological research (*n* = 4, 28.58%). The years of publication ranged from 2010 to 2022, with the most articles published in 2018 and 2020 (three articles each, or 21.42%). The most frequent data source was MEDLINE (*n* = 6, 42.88%), and Canada (*n* = 5, 35.74%) had the largest number of studies of any country of origin. The research’s language was predominantly English (*n* = 12, 85.72%) (Table 1).

**Table 1:** Main characteristics of articles included in the review (São Paulo, SP, Brazil, 2026).

Variables	Categories	Results	
		*N*	(%)
Year of publication	2010	1	7.15%
	2013	1	7.15%
	2015	1	7.15%
	2016	1	7.15%
	2017	1	7.15%
	2018	3	21.42%
	2020	3	21.42%
	2021	1	7.15%
	2022	2	14.26%
Data sources	Medline	6	42.88%
	References	3	21.42%
	VHL	1	7.14%
	Google Scholar	1	7.14%
	CAPES Theses and Dissertations	1	7.14%
	Global Observatory on Donation and Transplantation	1	7.14%
	Embase	1	7.14%
Country/continent of origin	Canada	5	35.74%
	Europe	2	14.28%
	Brazil	1	7.14%
	Australia	1	7.14%
	Spain	1	7.14%
	United States	1	7.14%
	Ireland	1	7.14%
	Poland	1	7.14%
	Trinidad and Tobago	1	7.14%
Language	English	12	85.72%
	Portuguese	1	7.14%
	Spanish	1	7.14%
Type/origin	Original article	11	78.58%
	Manual or guide	2	14.28%
	Review article	1	7.14%
Design/nature/approach	Quantitative research	7	50%
	Methodological research	4	28.58%
	Qualitative research	1	7.14%
	Secondary research	1	7.14%
	No information	1	7.14%

General characteristics of the included studies, presenting year of publication, data sources used, country or continent of origin, language, document type, and methodological design.

**Figure 2:**
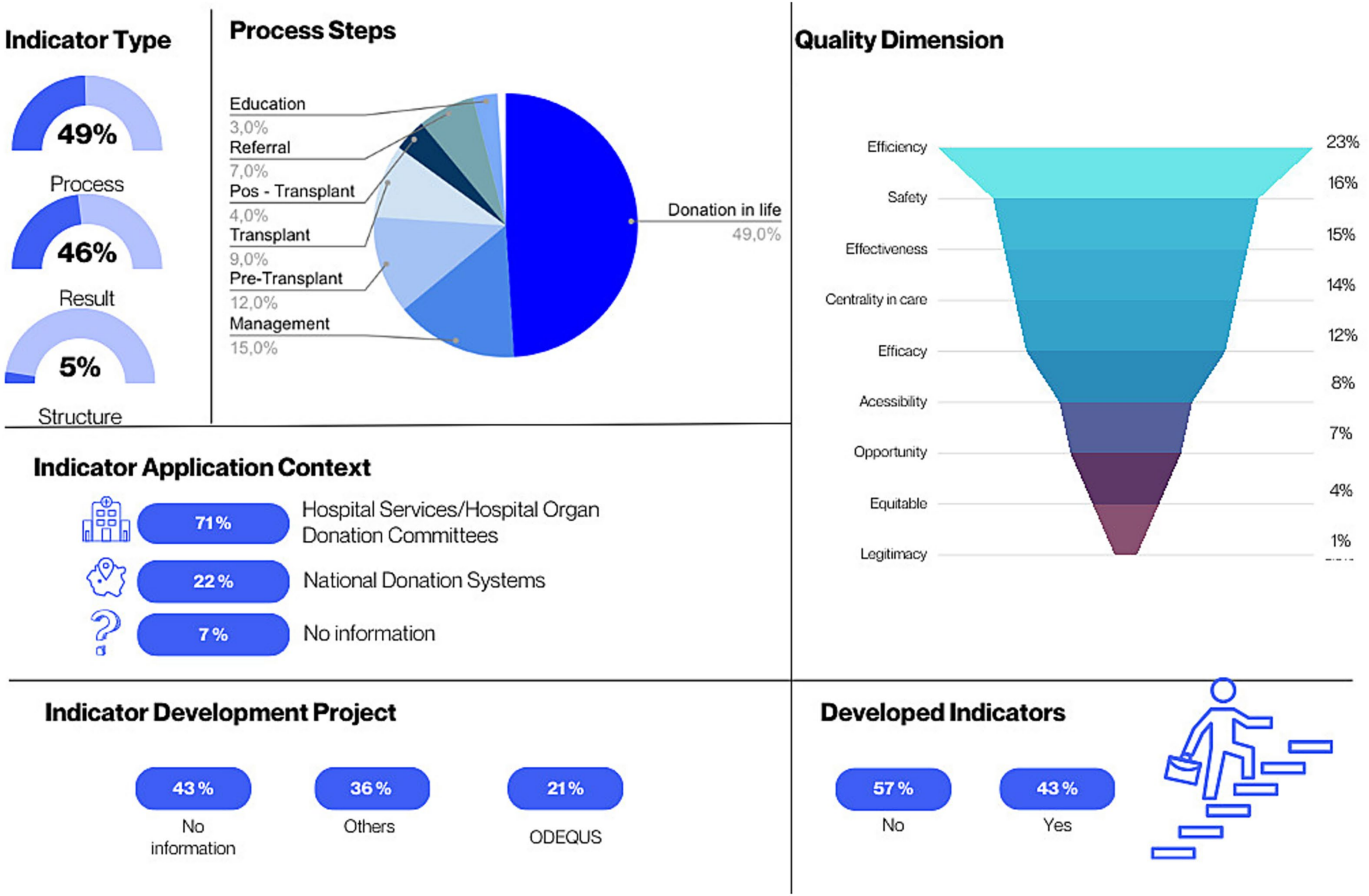
Characteristics of quality indicators in living kidney donation (São Paulo, SP, Brazil, 2026). Illustrative diagram of the typologies, quality dimensions, and process stages addressed in the study. The image organizes the key categories and highlights their interrelationships, as well as the different stages of assessment. It focuses on the variables that influence the performance and quality of the analyzed process.

Overall, 89 metrics were identified from the included studies. When analyzed, these metrics were unified into 74 indicators (15, 20–32). In Figure 2, it can be observed that most indicators are classified as process (*n* = 36; 49%), followed by outcome (*n* = 34; 46%) and structure (*n* = 4; 5%). The most common application context was hospital services/hospital committees of agencies (*n* = 10, 71.42%). With regard to the quality dimensions, most indicators are categorized under efficiency (*n* = 17; 23%), followed by safety (*n* = 12; 16%) and effectiveness (*n* = 11; 15%). The remaining dimensions include person-centeredness (*n* = 10; 14%), efficacy (*n* = 9; 12%), accessibility (*n* = 6; 8%), opportunity (*n* = 5; 7%), equity (*n* = 3; 4%), and legitimacy (*n* = 1; 1%) (Figure 2).

The indicators are related to different stages of the donation process, namely: living donation (*n* = 36, 49%); management (*n* = 11, 15%); pre-transplant (*n* = 9, 12%); transplant (*n* = 7, 9%); referral (*n* = 5, 7%); post-transplant (*n* = 3, 4%); education (*n* = 2, 3%); and waiting list (*n* = 1, 1%) (15, 20–32).

The most frequently cited indicators in this review were reported in three studies each. These include: [1] the annual number of preemptive kidney transplants from living donors, which measures the volume of transplant procedures performed before the initiation of dialysis and serves as a proxy for early access to transplantation and timely clinical management (15, 25, 29); [2] quality of life, which captures the well-being of donors and recipients across the pre-donation, perioperative, and post-transplant periods, encompassing physical and mental health, functional capacity, social reintegration, education, and socioeconomic conditions (15, 28, 30); and [3] the annual number of kidney transplants from living donors, an indicator that reflects the overall activity level of living-donor transplantation within a program or health system and is commonly used to monitor service capacity, trends in donation, and institutional performance (29, 30, 32).

**Figure 3:**
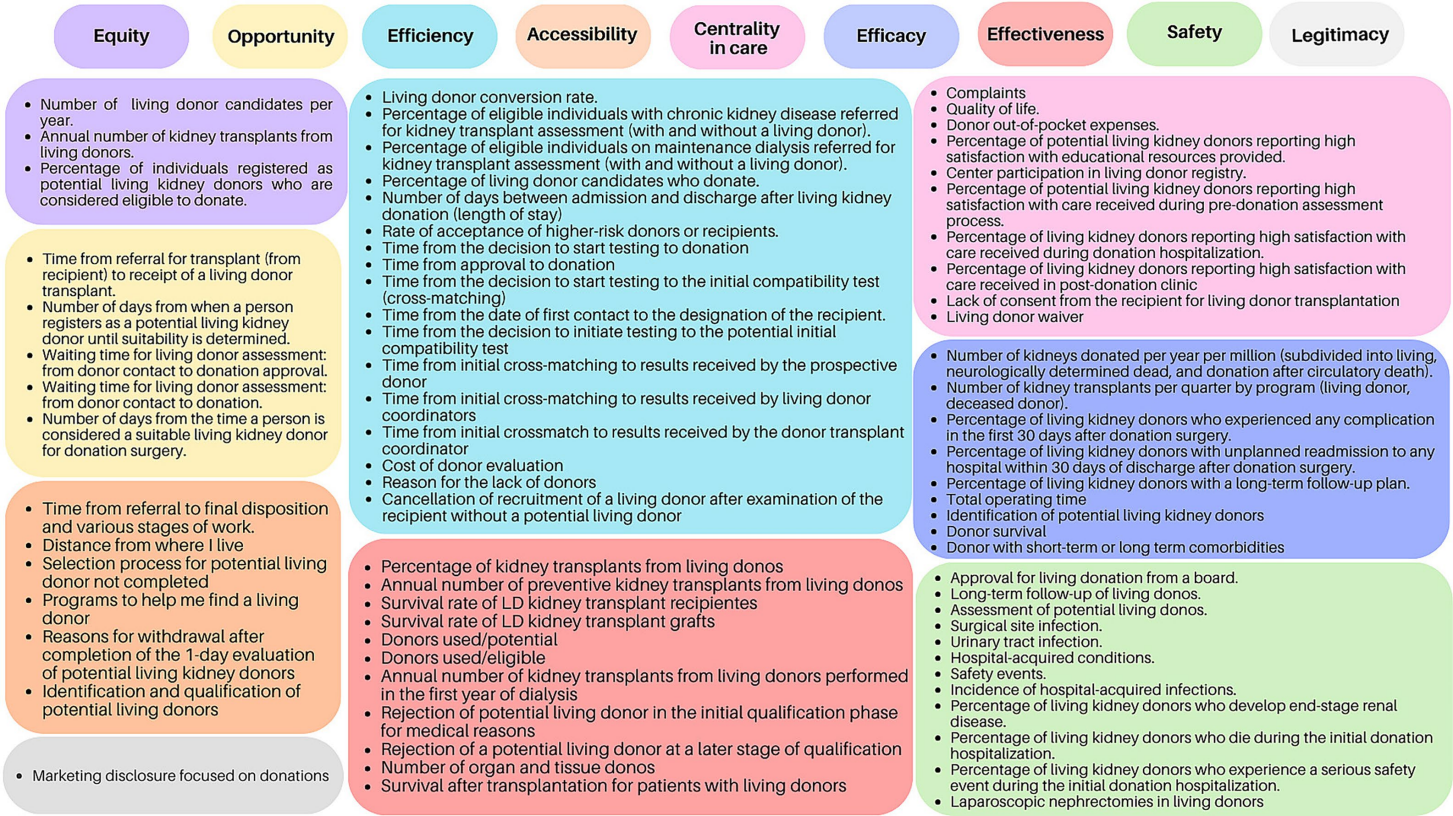
Quality indicators in living kidney donation (*n* = 74) (São Paulo, SP, Brazil, 2026). Figure depicting the quality dimensions, visually organized into color-coded quadrants, each containing the full names of the indicators associated with that dimension.

In addition to being quantified and categorized according to quality dimensions, the indicators were visually displayed in color-coded quadrants, with each quadrant representing one quality dimension and listing all corresponding indicators in full (Figure 3).

Regarding the indicator sheets, only 34% of the indicators (*n* = 25) had a complete and detailed specification, whereas 66% (*n* = 49) did not provide this information. Furthermore, 30% of the indicators were validated, while the remaining 70% lacked evidence of validity. Only 9% (*n* = 7) included a comparability standard, whereas 91% (*n* = 67) did not report any such standard (Supplementary material 1).

## Discussion

The findings of this scoping review reveal a fragmented landscape in the measurement of quality in living kidney donation. The lack of standardization in nomenclature, definitions, and methodological specifications across indicators suggests that the field has evolved in an uncoordinated manner, with different institutions and research groups adopting local solutions rather than converging toward unified quality-assessment practices. The heterogeneity observed across definitions, formulas, data sources, and reporting structures reflects not only methodological inconsistencies but also the absence of an international framework governing indicator development and use. This fragmentation limits the potential for comparison across programs and prevents the establishment of benchmarks that could strengthen oversight, performance evaluation, and quality improvement (20–33).

Despite this variability, the indicators identified in the literature reflect domains that are central to the functioning of living kidney donation programs. The predominance of process measures highlights the emphasis placed on operational efficiency and workflow optimization in this field, while the presence of outcome indicators demonstrates continued attention to donor and recipient well-being. However, the scarcity of indicators linked to broader dimensions of quality, particularly equity and legitimacy, suggests that essential aspects of the donation experience remain poorly assessed. Equity, for example, is a cornerstone of ethical transplantation practice, yet few measures address whether access to donation and transplantation is distributed fairly across different population groups. Similarly, legitimacy, which encompasses societal trust and alignment with community values, is rarely operationalized despite being central to public acceptance of donation systems (11–13, 16).

The limited methodological rigor of many indicators further constrains their usefulness. Few include detailed specifications, validation evidence, or comparability standards, which reduces their reliability in evaluation and quality assurance processes. Without clear definitions and validated measurement strategies, indicators risk being inconsistently applied or improperly interpreted. The literature corroborates this concern: previous reviews emphasize the need for transparent and standardized processes for indicator development, reinforcing that incomplete or poorly defined measures undermine the ability of health systems to engage in effective monitoring and improvement efforts (34). Addressing these methodological gaps is therefore essential to strengthening both the scientific and practical foundations of quality assessment in living kidney donation.

The three indicators most frequently cited in the literature illustrate both the strengths and limitations of current measurement practices. Metrics such as the annual number of preemptive living-donor transplants and the annual number of living-donor kidney transplants capture system activity and offer insight into program capacity and transplantation practices. However, these volume-based indicators provide limited information about the quality of the underlying processes or their consequences for donors and recipients. Their interpretability is constrained by the absence of expected standards, risk adjustment, and linkages to clinical outcomes, which restrict their ability to guide policy or compare performance across settings (15, 29, 32). The quality-of-life indicator, although clinically meaningful and aligned with patient-centered care, also suffers from a lack of specification and validation, making it difficult to determine how and when it should be measured or which instrument should be used (15, 28, 30). Together, these indicators underscore the dual challenge faced by the field: the need for both conceptual relevance and methodological precision.

The geographic distribution of the literature also merits attention. The predominance of studies from Canada suggests that certain health systems have advanced further in documenting and evaluating living kidney donation practices. Canada’s long-standing investment in donation programs and quality systems likely contributes to this leadership, offering opportunities for other countries to learn from established experiences (23). At the same time, the uneven distribution of studies across regions highlights the need for broader international engagement, particularly in low- and middle-income countries, where living donation often plays a critical role in transplant activity.

Another important finding is the limited representation of quality-assessment tools derived from established international frameworks, such as the Organ Donation European Quality System (ODEQUS). Although ODEQUS provides detailed and reproducible indicators for multiple forms of donation, including living donation, these tools appear to be underutilized in the literature analyzed. Wider adoption of frameworks like ODEQUS could facilitate methodological alignment across regions, enhance the comparability of indicators, and improve the reproducibility of quality assessments (21, 22, 27, 35). This gap also reinforces the need for future collaborative efforts to adapt and harmonize international frameworks to diverse healthcare contexts.

Overall, the results point to the necessity of developing more comprehensive and methodologically robust indicators that reflect all dimensions of quality in living kidney donation (36). This includes greater attention to equity, legitimacy, accessibility, and other underrepresented dimensions that shape donor and recipient experiences but are seldom captured in existing measurement tools. Strengthening the conceptual and methodological foundations of indicators is essential to ensuring that quality assessments can meaningfully guide quality improvement, resource allocation, and policy development.

Finally, the persistent variation in indicator definitions and the scarcity of technically mature measures underscore the importance of establishing a coordinated global effort to standardize quality indicators. Such an initiative would benefit from the participation of stakeholders across the transplant community, including healthcare professionals, donors, recipients, policymakers, and researchers. A harmonized set of well-defined, validated, and comparable indicators would provide a stronger foundation for evaluating performance, improving care processes, and promoting safe, effective, and equitable living-donor kidney transplantation. Recognizing these gaps is a necessary step toward building a more consistent and reliable quality framework for the field, one that supports both clinical practice and evidence-informed policy (11–13, 37, 38).

## Conclusion

There is significant variation in the nomenclature and definitions of quality indicators for living kidney donation. A deficit was identified in the availability of information on indicators, including their definitions, validations, calculation formulas, explanations of terms, quality dimensions, types, standards, and sources, among others. Most studies present quality indicators but do not associate them with a quality benchmark. The most frequently cited quality indicators for living kidney donation were quality of life, annual pre-emptive kidney transplants from living donors, and annual kidney transplants from living donors.
