# Supplementary material for: Quality indicators in living kidney donation for transplantation: a scoping review
Source: Front Med (Lausanne). 2026 May 8;13:1698289. doi: 10.3389/fmed.2026.1698289 (PMC13194424; doi:10.3389/fmed.2026.1698289)
Supplement: Supplementary file 1 [file Supplementary_file_1.DOCX]

**Supplementary Material 1**

**Document 1.Search terms and strategies.**

| **Descriptors/keyword used in the search strategy** | | |
| --- | --- | --- |
| **DeCS (Health Sciences Descriptors)** | **MeSH (Medical Subject Headings )** | **Keywords** |
| Doadores Vivos  Donadores Vivos  Rim  Riñón | Quality Indicators, Health Care  Kidney Transplantation  Living Donors  Health Status Indicators  Kidney | Quality  Qualidade  Calidad  Indicadores  Transplante de rim  Indicadores de qualidade  Doação  Transplante  Doação renal  Doação de rins  Transplante de rins  Doação em vida  Quality indicators  Quality metrics  Indicator  Indicators  Healthcare Quality Indicator Healthcare Quality Indicators  Indicator, Healthcare Quality Indicators, Healthcare Quality Quality Indicator, Healthcare Health Metrics  Health Metric  Metrics  Metrics, Health  Health Status Indicator Health Status Index  Health Status Indices  Health Status Indexes  Health Risk Appraisal  Health Risk Appraisals  Donors, Living  Donor, Living  Living Donor  Living Donors  Living kidney Donors  kidney Donors  Kidney donation  Renal Transplantation  Renal Transplantations  Transplantations, Renal  Transplantation, Renal  Grafting, Kidney  Kidney Grafting  Transplantation, Kidney  Kidney Transplantations  Transplantations, Kidney |
|  |  |  |
|  |  |  |
|  |  |  |
|  |  |  |
|  |  |  |
|  |  |  |
|  |  |  |
|  |  |  |
|  |  |  |
|  |  |  |
| **The search was conducted from March 9 to May 25, 2023** | | |
| **Database: MEDLINE (Pubmed)** | | |
| **Search strategy** | | |
| *((((((("Quality Indicators, Health Care"[Mesh] OR "Healthcare Quality Indicator" OR "Healthcare Quality Indicators" OR "Indicator, Healthcare Quality" OR "Indicators, Healthcare Quality" OR "Quality Indicator, Healthcare" OR "Health Metrics" OR "Health Metric" OR "Metrics, Health")) OR (("quality indicators, health care"[MeSH Terms] OR "Healthcare Quality Indicator"[All Fields] OR "Healthcare Quality Indicators"[All Fields] OR "Health Metrics"[All Fields] OR "Health Metric"[All Fields] OR "quality metric"[Title/Abstract] OR "quality metrics"[Title/Abstract]) OR ("Health Status Indicators"[MeSH Terms] OR "Health Status Indicator"[All Fields] OR "Health Status Index"[All Fields] OR "Health Status Indices"[All Fields] OR "Health Status Indexes"[All Fields] OR "Health Risk Appraisal"[All Fields] OR "Health Risk Appraisals"[All Fields]))) OR (("quality indicators"[All Fields] OR "quality metrics"[All Fields]))) AND (("Kidney"[Mesh]) AND (("Living Donors"[Mesh] OR "Donors, Living" OR "Donor, Living" OR "Living Donor")))) OR ((((("Quality Indicators, Health Care"[Mesh] OR "Healthcare Quality Indicator" OR "Healthcare Quality Indicators" OR "Indicator, Healthcare Quality" OR "Indicators, Healthcare Quality" OR "Quality Indicator, Healthcare" OR "Health Metrics" OR "Health Metric" OR "Metrics, Health")) OR (("quality indicators, health care"[MeSH Terms] OR "Healthcare Quality Indicator"[All Fields] OR "Healthcare Quality Indicators"[All Fields] OR "Health Metrics"[All Fields] OR "Health Metric"[All Fields] OR "quality metric"[Title/Abstract] OR "quality metrics"[Title/Abstract]) OR ("Health Status Indicators"[MeSH Terms] OR "Health Status Indicator"[All Fields] OR "Health Status Index"[All Fields] OR "Health Status Indices"[All Fields] OR "Health Status Indexes"[All Fields] OR "Health Risk Appraisal"[All Fields] OR "Health Risk Appraisals"[All Fields]))) OR (("quality indicators"[All Fields] OR "quality metrics"[All Fields]))) AND ((("Living Donors"[Mesh] OR "Donors, Living" OR "Donor, Living" OR "Living Donor")) AND (("Kidney Transplantation"[Mesh] OR "Renal Transplantation" OR "Renal Transplantations" OR "Transplantations, Renal" OR "Transplantation, Renal" OR "Grafting, Kidney" OR "Kidney Grafting" OR "Transplantation, Kidney" OR "Kidney Transplantations" OR "Transplantations, Kidney"))))) OR ((((((("Quality Indicators, Health Care"[Mesh] OR "Healthcare Quality Indicator" OR "Healthcare Quality Indicators" OR "Indicator, Healthcare Quality" OR "Indicators, Healthcare Quality" OR "Quality Indicator, Healthcare" OR "Health Metrics" OR "Health Metric" OR "Metrics, Health")) OR (("quality indicators, health care"[MeSH Terms] OR "Healthcare Quality Indicator"[All Fields] OR "Healthcare Quality Indicators"[All Fields] OR "Health Metrics"[All Fields] OR "Health Metric"[All Fields] OR "quality metric"[Title/Abstract] OR "quality metrics"[Title/Abstract]) OR ("Health Status Indicators"[MeSH Terms] OR "Health Status Indicator"[All Fields] OR "Health Status Index"[All Fields] OR "Health Status Indices"[All Fields] OR "Health Status Indexes"[All Fields] OR "Health Risk Appraisal"[All Fields] OR "Health Risk Appraisals"[All Fields]))) OR (("quality indicators"[All Fields] OR "quality metrics"[All Fields]))) AND ((("Kidney Transplantation"[Mesh] OR "Renal Transplantation" OR "Renal Transplantations" OR "Transplantations, Renal" OR "Transplantation, Renal" OR "Grafting, Kidney" OR "Kidney Grafting" OR "Transplantation, Kidney" OR "Kidney Transplantations" OR "Transplantations, Kidney")) OR (("Living Donors"[Mesh] OR "Donors, Living" OR "Donor, Living" OR "Living Donor")))) AND ((((("Quality Indicators, Health Care"[Mesh] OR "Healthcare Quality Indicator" OR "Healthcare Quality Indicators" OR "Indicator, Healthcare Quality" OR "Indicators, Healthcare Quality" OR "Quality Indicator, Healthcare" OR "Health Metrics" OR "Health Metric" OR "Metrics, Health")) OR (("quality indicators, health care"[MeSH Terms] OR "Healthcare Quality Indicator"[All Fields] OR "Healthcare Quality Indicators"[All Fields] OR "Health Metrics"[All Fields] OR "Health Metric"[All Fields] OR "quality metric"[Title/Abstract] OR "quality metrics"[Title/Abstract]) OR ("Health Status Indicators"[MeSH Terms] OR "Health Status Indicator"[All Fields] OR "Health Status Index"[All Fields] OR "Health Status Indices"[All Fields] OR "Health Status Indexes"[All Fields] OR "Health Risk Appraisal"[All Fields] OR "Health Risk Appraisals"[All Fields]))) OR (("quality indicators"[All Fields] OR "quality metrics"[All Fields]))) AND ((("Kidney Transplantation"[Mesh] OR "Renal Transplantation" OR "Renal Transplantations" OR "Transplantations, Renal" OR "Transplantation, Renal" OR "Grafting, Kidney" OR "Kidney Grafting" OR "Transplantation, Kidney" OR "Kidney Transplantations" OR "Transplantations, Kidney")) OR (("Living Donors"[Mesh] OR "Donors, Living" OR "Donor, Living" OR "Living Donor"))))) AND ("Kidney"[Mesh]))) OR (((("Living Donors"[Mesh] OR "Living Donors") AND ("Kidney"[Mesh] OR Kidney)) AND ("Quality Indicators, Health Care" OR Metrics)) AND ("Quality Indicators, Health Care" OR Metrics))*  *No filters or time limits.* | | |
|  |  |  |
|  |  |  |
|  |  |  |
|  |  |  |
|  |  |  |
|  |  |  |
|  |  |  |
|  |  |  |
| **Database: CINAHL (EBSCO)** | | |
| **Search strategy** | | |
| *(((MH "Living Donors") OR "Living Donors") AND ((MH "kidney") OR "kidney")) AND (Indicator OR Indicators)*  *No filters or time limits.* | | |
|  |  |  |
|  |  |  |
|  |  |  |
|  |  |  |
|  |  |  |
|  |  |  |
|  |  |  |
|  |  |  |
| **Database: EMBASE (Elsevier)** | | |
| **Search strategy** | | |
| *('living donor'/exp OR 'living donor') AND ('kidney'/exp OR kidney) AND ('indicators'/exp OR indicators)*  *No filters or time limits.* | | |
|  |  |  |
|  |  |  |
|  |  |  |
|  |  |  |
|  |  |  |
|  |  |  |
|  |  |  |
|  |  |  |
| **Database: SCOPUS (Elsevier)** | | |
| **Search strategy** | | |
| *("Living Donors" AND Kidney) AND (Quality AND Indicators)*  *No filters or time limits.* | | |
|  |  |  |
|  |  |  |
|  |  |  |
|  |  |  |
|  |  |  |
|  |  |  |
|  |  |  |
|  |  |  |
| **Database: BVS (Bireme)** | | |
| **Search strategy** | | |
| *((“Doadores Vivos” OR “Living Donors” OR “Donadores Vivos”) AND (Rim OR Kidney OR Riñón)) AND ((Qualidade OR Quality OR Calidad) AND (Indicadores OR Indicators))*  *No filters or time limits.* | | |
|  |  |  |
|  |  |  |
|  |  |  |
|  |  |  |
|  |  |  |
|  |  |  |
|  |  |  |
|  |  |  |
| **Database: WEB OF SCIENCE** | | |
| **Search strategy** | | |
| *("Living Donors" AND Kidney) AND (Quality AND Indicators)* | | |
| **Grey literature: Google Scholar** | | |
| **Search strategy** | | |
| (("Living Donors" AND Kidney) AND "Quality indicators")  Filter: Quotes removed. | | |
|  |  |  |
|  |  |  |
|  |  |  |
|  |  |  |
|  |  |  |
|  |  |  |
|  |  |  |
|  |  |  |
| **Grey literature: websites** | | |
| **website: OpenGrey (GreyNet) - http://www.opengrey.eu/** | | |
| **Search strategy** | | |
| Description of the search: Performed in the Search Bar.  Full Search strategy: #1 “kidney” AND “indicators”  #2 “Living kidney Donors”  #3 “kidney Donors”  #4 “Quality indicators” AND “Kidney donation” | | |
| **website: Theses and dissertations CAPES (BRA) - https://catalogodeteses.capes.gov.br/catalogo-teses/#!/** | | |
| **Search strategy** | | |
| Description of the search: Performed in the Search Bar.  Full Search strategy: #1 “Indicadores” AND "transplante de rim"  #2 ”Indicadores de qualidade" AND “doação”  #3. "Indicadores de qualidade" AND “transplante” | | |
| **website: Global ETD Search (previously: Union Catalog) - http://search.ndltd.org/index.php** | | |
| **Search strategy** | | |
| Description of the search: Performed in the Search Bar.  Full Search strategy: #1 "quality indicators" AND “kidney” | | |
| **website: Open Access Theses and Dissertations - https://oatd.org/oatd/search?q=+quality+indicators+AND+kidney&form=basic** | | |
| **Search strategy** | | |
| Description of the search: Performed in the Search Bar.  Full Search strategy: #1 "quality indicators" AND “kidney” | | |
| **website: The Transplantation Society (TTS) - https://tts.org/** | | |
| **Search strategy** | | |
| Description of the search: Performed in the Search Bar.  Full Search strategy: #1 “Living Donors” #2 “Kidney” #3 “Quality” #4 “Indicators”  #5 “Living Donors” AND “Kidney”  #6 “Quality” AND “Indicators”  #7 (Living Donors AND Kidney) AND (Quality AND Indicators) | | |
| **website: Global Observatory on Donation and Transplantation - http://www.transplant-observatory.org/** | | |
| **Search strategy** | | |
| Description of the search: Performed in the Search Bar.  Full Search strategy: #1 “Living Donors” #2 “Kidney” #3 “Quality” #4 “Indicators”  #5 “Living Donors” AND “Kidney”  #6 “Quality” AND “Indicators”  #7 (Living Donors AND Kidney) AND (Quality AND Indicators) | | |
| **website: Organización Nacional de Trasplantes (ONT) - http://www.ont.es/Paginas/Home.aspx** | | |
| **Search strategy** | | |
| Searched in the scientific articles tab, which is separated by year and the total number of studies published. A title-by-title analysis. | | |
| **website: Sociedad de Transplante de América Latina y el Caribe (STALYC) - https://stalyc.net/** | | |
| **Search strategy** | | |
| Description of the search: Performed in the Search Bar.  Full Search strategy: #1 “Living Donors” #2 “Kidney” #3 “Quality” #4 “Indicators”  #5 “Living Donors” AND “Kidney”  #6 “Quality” AND “Indicators”  #7 (Living Donors AND Kidney) AND (Quality AND Indicators) | | |
| **website:European Society for Organ Transplantation (ESOT) - https://esot.org/** | | |
| **Search strategy** | | |
| Description of the search: Performed in the Search Bar.  Full Search strategy: #1 “Living Donors” #2 “Kidney” #3 “Quality” #4 “Indicators”  #5 “Living Donors” AND “Kidney”  #6 “Quality” AND “Indicators”  #7 (Living Donors AND Kidney) AND (Quality AND Indicators) | | |
| **website:National Institute for Health and Care Excellence -https://www.nice.org.uk/** | | |
| **Search strategy** | | |
| Description of the search: Performed in the Search Bar.  Full Search strategy: #1 “Living Donors” #2 “Kidney” #3 “Quality” #4 “Indicators”  #5 “Living Donors” AND “Kidney”  #6 “Quality” AND “Indicators”  #7 (Living Donors AND Kidney) AND (Quality AND Indicators) | | |
| **website:Agency for Healthcare Research and Quality -https://www.ahrq.gov/** | | |
| **Search strategy** | | |
| Description of the search: Performed in the Search Bar.  Full Search strategy: #1 “Living Donors” #2 “Kidney” #3 “Quality” #4 “Indicators”  #5 “Living Donors” AND “Kidney”  #6 “Quality” AND “Indicators”  #7 (Living Donors AND Kidney) AND (Quality AND Indicators)  It has a field for advanced searches. | | |
| **website:Healthcare Quality Improvement Partnership -https://www.hqip.org.uk/** | | |
| **Search strategy** | | |
| Description of the search: Performed in the Search Bar.  Full Search strategy: #1 “Living Donors” #2 “Kidney” #3 “Quality” #4 “Indicators”  #5 “Living Donors” AND “Kidney”  #6 “Quality” AND “Indicators”  #7 (Living Donors AND Kidney) AND (Quality AND Indicators) | | |
| **website:National Quality Forum -https://www.qualityforum.org/Home.aspx** | | |
| **Search strategy** | | |
| Description of the search: Performed in the Search Bar.  Full Search strategy: #1 "Living donors"  #2. "Kidney transplantation" | | |
| **website:International Society for Quality in Health Care (ISQua) -https://isqua.org/** | | |
| **Search strategy** | | |
| Description of the search: Performed in the Search Bar.  Full Search strategy: #1 "Living donors"  #2. "Kidney transplantation"  #3. “Living kidney Donors” | | |
| **website:ANVISA – National Health Surveillance Agency (BRA) -https://www.gov.br/anvisa/pt-br** | | |
| **Search strategy** | | |
| Description of the search: Performed in the Search Bar.  Full Search strategy: #1 "indicadores de qualidade" AND "Doação renal"  #2 "Indicadores de qualidade" AND “Doação de rins"  #3 "Indicadores de qualidade" AND "Transplante de rins"  #4 "Transplante de rins"  #5 "Doação em vida" | | |
| **website: PROQUALIS – Collaborating Centre for the Quality of Care and Patient Safety**  **- https://proqualis.net/** | | |
| **Search strategy** | | |
| Description of the search: Performed in the Search Bar.  Full Search strategy: #1 “Transplante” #2 "Doação em vida" #3 “Doação de rins" | | |

**Document 2. Summary table of all included studies and their characteristics (n=14).**

| **Authors/**  **Year/Source** | **Objective** | **Method** | **Scenario/Country of origin** | **Quality indicators** |
| --- | --- | --- | --- | --- |
| [1]Cienfuegos et al., / 2016 / Medline | Evaluating health quality indicators between 2013 and 2014 in the area of kidney transplantation in a service. | Quantitative / Descriptive | Spain | 1- Percentage of kidney transplants from living donors.  2- Laparoscopic nephrectomy in living donors. |
| [2] Elcock - Straker et al., / 2022 / Medline | Analyzed the 15-year experience of the public program in terms of transplant results and acquisition quality at the main deceased donor acquisition hospital, using DOPKI and ODEQUS quality indicators. | Quantitative /  Descriptive | Trindad E Tobago | 1-Survival rate of kidney transplant recipients LD  2- Kidney transplant graft survival rate LD  3- Donors used/potential donors  4- Donors used/eligible donors |
| [3]European Committee (Partial Agreement) on Organ Transplantation/ 2022 / Global Observatory on Donation and Transplantation | The development of instruments to measure quality has been essential in turning this concern into a way of working. | Guideline | Europe | 1- Approval for a living donation from a board  2- Participation of the center in the registry of living donors  3- Identifying potential living kidney donors  4-Long-term follow-up of living donors  5- Evaluation of potential living donors |
| [4] Glavinovic, T et al.,/ 2021 / PUBMED | Identify, categorize and evaluate the strengths and weaknesses of kidney transplant quality indicators currently used across Canada | Methodological / Descriptive | Canada | 1- Number of kidneys donated per year per million (subdivided into living, neurological determination of death and donation after circulatory death)  2- Number of kidney transplants per quarter per program (living donor, deceased donor)  3-Percentage of eligible individuals with chronic kidney disease referred for kidney transplant evaluation (with or without living donor)  4- Percentage of eligible individuals on maintenance dialysis referred for kidney transplant evaluation (with and without living donor)  5- Percentage of prospective living donors who donate  6- Time from transplant referral (from the recipient) to receiving a living donor transplant  7-Waiting time for live donor evaluation: from donor contact to donation approval  8- Waiting time for live donor evaluation: from donor contact to donation  9- Number of living donor candidates per year |
| [5] Schaffhausen C.R et al., / 2018 / Medline | Collect data on participant characteristics and priorities for important information for choosing a transplant program. | Quantitative /  Exploratory / Descriptive | United States | 1 - Distance from where I live  2- Programs to help me find a living donor  3-Survival after transplantation for patients with a living donor |
| [6] Toussaint, N.D et al., / 2015 / EMBASE | It describes a summary of the implementation of renal KPIs and subsequent trends in clinical practice across Victorian renal services. | Quantitative / Descriptive | Australia | 1-Proportion of new live donor transplants that are pre-emptive |
| [7] Treviso et al., / 2010 / TESES E DISSERTAÇÕES CAPES | To identify the main quality indicators for a kidney transplant service in the opinion of professionals and patients and to find out how important the dimensions of quality are for these patients. To analyze the importance attributed by professionals to a list of care and administrative indicators related to the institution and the Renal Transplant Service. | Quantitative / Cross-sectional | Brazil | 1-Number of organ and tissue donors  2-Donation-focused marketing  3-Reason for lack of donors |
| [8] Martí Manyalich, et al., / 2013 / REFERÊNCIAS | Once the ODEQUS quality system has been applied at hospital level, it will be possible to standardize the organ donation process, creating a methodology to evaluate the performance of organ harvesting and defining improvement strategies to increase the efficiency of transplant systems in Europe. | Methodological / Exploratory | Europe | 1- Approval for living donation from a council  2- Participation of the centre in living donors registry  3- Identification of potential kidney living donors  4- Long-term follow-up of living donors  5- Evaluation of potential living donors |
| [9] Brett KE et al., / 2018 / REFERÊNCIAS | The aim was to find out what patients, doctors and program administrators think about quality care for kidney transplant patients. | Qualitative / Exploratory / Descriptive | Canada | 1- Time reference to final disposal and various stages of work  2- Time from registration to testing, from testing to clinical visits, from clinical visit to approval, approval and donation  3-Donor survival  4-Donor with short or long-term comorbidities  5- Cost of evaluating donors  6- Donors who start and finish the process  7- Donor's own expenses  8- Incidence of hospital-acquired infections  9- Quality of life  10- Complaints  11- Acceptance rate of higher risk donors or recipients |
| [10] Habbous, Steven et al., / 2020 / BVS Virtual Health Library | Develop consensus on the main terms and indicators that can be used to measure the efficiency with which a transplant center evaluates people interested in becoming a living kidney donor. | Methodological / Exploratory | Canada | 1- Time from the decision to start the test to donation  2- Time from the decision to start the test to the initial compatibility test (cross-matching)  3- Time from the date of first contact to the appointment of the recipient  4- Time from the decision to start the test to the potential initial compatibility test  5-Time from the initial cross-check to the results received by the prospective donor  6- Time from initial cross-check to results received by living donor coordinators  7- Time from the initial cross-check to the results being received by the donor's transplant coordinator  8- Time from approval to donation  9- Total cost of health care for the evaluation of the candidate living donor  10- Annual number of living donor kidney transplants  11-Annual number of preventive kidney transplants from living donors  12-Annual number of living donor kidney transplants performed in the first year of dialysis |
| [11] Knoll et al./ 2020 / Medline | To define which of the many proposed quality indicators are important and should be measured, we held a Canadian consensus workshop involving the main stakeholders in kidney transplantation. | Methodological/ Descriptive | Canada | 1- Percentage of individuals registered as potential living kidney donors who are considered fit to donate  2- Number of living donor kidney transplants performed  3-Number of days from when the person registers as a potential living kidney donor to when suitability is determined  4-Number of days from the time the person is considered a suitable living kidney donor for surgery  5-Number of days between admission and discharge after living kidney donation (length of stay)  6- Percentage of living kidney donors who develop end-stage renal disease  7- Percentage of living kidney donors who die during the initial hospitalization for donation  8- Percentage of living kidney donors who suffer a serious safety event during the initial hospitalization period for donation  9- Percentage of living kidney donors who had a complication in the first 30 days after donation surgery  10-Percentage of living kidney donors with unplanned readmission to any hospital within 30 days of discharge after donation surgery  11- Percentage of living kidney donors with a long-term follow-up plan  12- Percentage of potential living kidney donors who report a high level of satisfaction with the educational resources provided  13- Percentage of potential living kidney donors who report a high level of satisfaction with the care received during the pre-donation assessment process  14-Percentage of living kidney donors who report a high level of satisfaction with the care received during the donation hospitalization  15- Percentage of living kidney donors who report a high level of satisfaction with the care received in the post-donation clinic  16- Percentage of living kidney donors reporting excellent HRQoL after donation |
| [12] Brett KE et al. / 2018 / PUBMED | The aim was to systematically identify, describe and characterize the impact of quality of care metrics in the field of solid organ transplantation. | Secondary Research / Systematic Review | Canada | 1- Proportion of patients with pre-emptive kidney transplantation  2- Acceptance rate of higher risk donors or recipients  3-Living donor conversion rate  4- Total operating time  5-Surgical site infection  6- Urinary tract infection  7- Hospital-acquired conditions  8-Security events  9- Quality of life |
| [13] Judi M et. al/ 2017 / REFERÊNCIAS | It was a quality improvement project that sought out all potential donors deemed suitable after a screening questionnaire, and participated in a comprehensive 1-day assessment, including all the investigations that were previously implemented in multiple clinic visits | Quantitative / Cross-sectional | Ireland | 1-Reasons for withdrawal after completion of the 1-day evaluation of the potential living kidney donor  2- Living donor (LD) transplant rates |
| [14] M. Hermanowicz et. al / 2020 / GOOGLE SHOLAR | Living donor accepted and kidney transplant performedThe program aims to build and develop a high-quality donation system at hospital and national level. | Quantitative / Descriptive | Poland | 1- Canceling the recruitment of a living donor after examining the recipient without a potential living dono  2- Lack of recipient consent for living donor transplants  3- Identification and qualification of potential living donors  4-Rejection of a potential living donor in the initial qualification phase for medical reasons  5- Rejection of a potential living donor at a later qualifying stage  6- Living donor renunciation  7-Potential living donor selection process not completed  8-Living donor accepted and kidney transplant performed |

**Document 3 - Characteristics of the quality indicators for living kidney donation included in the study (n=74).**

| **Indicator title** | **Type of Indicator** | **Quality Dimension** | **Validated Indicator** | **Indicator sheet** | **Indicator Standard** |
| --- | --- | --- | --- | --- | --- |
|  |  |  |  |  |  |
| Approval for a living donation from a board^3,8^ | Process | Safety | No | Yes | 100% |
| Participation of the center in the registry of living donors^3,8^ | Process | Patient-centredness | No | No | 100% |
| Long-term follow-up of living donors^3,8^ | Process | Safety | Yes | Yes | 100% |
| Time from the decision to start the test to donation ^9,10^ | Process | Efficiency | No | No | - |
| Living donor conversion rate^12^ | Process | Efficiency | No | No | - |
| Total operating time^12^ | Process | Efficacy | No | No | - |
| Percentage of eligible individuals with chronic kidney disease referred for kidney transplant evaluation (with and without living donor)^4^ | Process | Efficiency | Yes | No | - |
| Percentage of eligible individuals on maintenance dialysis referred for kidney transplant evaluation (with and without living donor)^4^ | Process | Efficiency | Yes | No | - |
| Time from approval to donation^10^ | Process | Efficiency | No | No | - |
| Time from transplant referral (from the recipient) to receiving a living donor transplant^4^ | Process | Opportunity | Yes | No | - |
| Time from transplant referral (from recipient) to receiptLiving donor evaluation waiting time: from donor contact to approval of a living donor transplant donation^4?^ | Process | Opportunity | Yes | No | - |
| Waiting time for live donor evaluation: from donor contact to donation^4?^ | Process | Opportunity | Yes | No | - |
| Time from the decision to start the test to the initial compatibility test (cross-matching)^10^ | Process | Efficiency | No | No | - |
| Time from the date of first contact to indication of the recipient^10^ | Process | Efficiency | Yes | No | - |
| Time from the decision to start the test to the potential initial compatibility test^10^ | Process | Efficiency | Yes | No | - |
| Time from the initial cross-check to the results received by the prospective donor^10^ | Process | Efficiency | Yes | No | - |
| Time from initial cross-check to results received by living donor coordinators^10^ | Process | Efficiency | Yes | No | - |
| Time from the initial cross-check to the results being received by the donor's transplant coordinator^10^ | Process | Efficiency | Yes | No | - |
| Number of living donor candidates per year^4^ | Process | Equitable | Yes | No | - |
| Identification of potential kidney living donors^3,8^ | Results | Efficacy | Yes | Yes | 20% |
| Evaluation of potential living donors^3,8^ | Results | Safety | Yes | Yes | 80% |
| Quality of life^9, 11, 12^ | Results | Patient-centredness | No | No | - |
| Cost of evaluating donors ^9,10^ | Results | Efficiency | Yes | No | - |
| Percentage of kidney transplants from living donors^1, 13^ | Results | Effectiveness | No | Yes | 10% |
| Annual number of pre-emptive kidney transplants from living donors^6, 10, 12^ | Results | Effectiveness | Yes | No | - |
| Annual number of living donor kidney transplants^10, 11, 14^ | Results | Equitable | Yes | No | - |
| % of living donor candidates who donate ^4,9^ | Results | Efficiency | Yes | No | - |
| Survival rate of kidney transplant recipients LD^2^ | Results | Effectiveness | No | Yes | - |
| Kidney transplant graft survival rate LD^2^ | Results | Effectiveness | No | Yes | - |
| Living donor laparoscopic nephrectomy^1^ | Results | Safety | No | Yes | 60% |
| Surgical site infection^12^ | Results | Safety | No | No | - |
| Urinary tract infection^12^ | Results | Safety | No | No | - |
| Hospital-acquired conditions^12^ | Results | Safety | - | No | - |
| Security events^12^ | Results | Safety | - | No | - |
| Donors used/potential^2^ | Results | Effectiveness | - | No | - |
| Donors used/eligible^2^ | Results | Effectiveness | - | No | - |
| Nº of kidneys donated per year per million (subdivided into living, neurological determination of death and donation after circulatory death)^4^ | Results | Efficacy | - | Yes | - |
| Number of kidney transplants per quarter per program (living donor, deceased donor)^4^ | Results | Efficacy | - | Yes | - |
| Annual number of living donor kidney transplants performed in the first year of dialysis^10^ | Results | Effectiveness | Yes | No | - |
| Acceptance rate of higher risk donors or recipients^9^ | Structure | Efficiency | - | No | - |
| Donor's own expenses^9^ | Process | Patient-centredness | - | No | - |
| Distance from where I live^5^ | Structure | Accessibility | Yes | No | - |
| Potential living donor selection process not completed^14^ | Process | Accessibility | No | No | - |
| Complaints^9^ | Process | Patient-centredness | - | No | - |
| Donation-focused marketing^7^ | Process | Legitimacy | - | No | - |
| Incidence of hospital-acquired infections^9^ | Result | Safety | - | No | - |
| Reason for lack of donors^7^ | Process | Efficiency | - | No | - |
| Programs to help me find a living donor ^5^ | Structure | Accessibility | No | No | - |
| Percent of individuals registered as potential living kidney donors who are considered fit to donate^11^ | Process | Equitable | Yes | Yes | - |
| Percent of living kidney donors who develop end-stage renal disease^11^ | Result | Safety | - | Yes | - |
| Percentage of living rim donors who die during the initial hospitalization for donation^11^ | Result | Safety | - | Yes | - |
| The percentage of living rim donors who suffer a serious safety event during the initial hospitalization period for donation^11^ | Result | Safety | - | Yes | - |
| Percentage of living kidney donors who had a complication in the first 30 days after donation surgery^11^ | Result | Efficacy | - | Yes | - |
| Percentage of living kidney donors with unplanned readmission to any hospital within 30 days of discharge after donation surgery ^11^ | Result | Efficacy | - | Yes | - |
| Percentage of living kidney donors with a long-term follow-up plan ^11^ | Process | Efficacy | - | Yes | - |
| Percentage of potential living kidney donors who report a high level of satisfaction with the educational resources provided^11^ | Results | Patient-centredness | - | Yes | - |
| Percentage of potential living kidney donors who report a high level of satisfaction with the care received during the pre-donation assessment process^11^ | Results | Patient-centredness | - | Yes | - |
| Percentage of living kidney donors who report a high level of satisfaction with the care received during the donation hospitalization  ^11^ | Result | Patient-centredness | - | Yes | - |
| Percentage of living kidney donors who report a high level of satisfaction with the care received in the post-donation clinic^11^ | Result | Patient-centredness | - | Yes | - |
| Reasons for withdrawal after completion of the 1-day evaluation of the potential living kidney donor^13^ | Process | Accessibility | - | No | - |
| Canceling the recruitment of a living donor after examining the recipient without a potential living donor^14^ | Process | Efficiency | - | No | - |
| Lack of recipient consent for living donor transplants^14^ | Process | Patient-centredness | - | No | - |
| Identification and qualification of potential living donors^14^ | Process | Accessibility | No | No | - |
| Rejection of a potential living donor in the initial qualification phase for medical reasons ^14^ | Process | Effectivenes | No | No | - |
| Rejection of a potential living donor at a later qualifying stage ^14^ | Process | Effectivenes | No | No | - |
| Living donor renunciation ^14^ | Process | Patient-centredness | No | No | - |
| Time reference to final disposal and various stages of work ^9^ | Process | Accessibility | Yes | Yes | - |
| Number of days from when the person registers as a potential living kidney donor to when suitability is determined ^11^ | Process | Opportunity | - | Yes | - |
| Number of days from the time the person is considered a suitable living kidney donor for surgery ^11^ | Process | Opportunity | - | Yes | - |
| Number of days between admission and discharge after living kidney donation (length of stay) ^11^ | Process | Efficiency | - | Yes | - |
| Number of organ and tissue donors ^7^ | Result | Effectiveness | No | No | - |
| Survival after transplantation for patients with a living donor ^5^ | Result | Effectiveness | No | No | - |
| Donor survival ^9^ | Result | Efficacy | - | No | - |
| Donor with short or long-term comorbidities ^9^ | Structure | Efficacy | - | No | - |

**Document 4 - Quality indicator sheets for living kidney donation, which contained the information needed for construction.**

| **Title** | Approval for inter-vivos donation by a council ^3, 8^ |
| --- | --- |
| **Justification** | To ensure the best outcome and safety for both the recipient and the living donor, all donors must obtain approval for donation from a multidisciplinary board with doctors from transplant-related medical specialties (i.e. nephrologists, hepatologists, Tx surgeons, immunologists) at the transplant centre. Their judgement must conform to the guiding principles established by the ethics committee assigned to each transplant centre. |
| **Dimension** | Security |
| **Formula** | Number of potential LD investigated and approved from a specific group/ total number of actual living donors x 100 |
| **Explanation of terms** | • LD: LD: Living kidney or liver donor  • Potential living donor: A legally competent person who has expressed their willingness to donate an organ while alive and is apparently suitable for living organ donation.  • Real living donor: an eligible living donor.  • in whom an operative incision was made with the intention of recovering organs for the purpose of transplantation or from whom at least one organ was recovered for the purpose of transplantation.  • Approved: Documented record of the specific group's approval (in the medical records department or in the group's specific minutes).  • Specific group: a multidisciplinary group independent of the recipient organisation, which aims to assess the donor (motivation, medical and socio-economic situation). |
| **Population** | All real living donors were approved during the period studied. |
| **Type** | Process |
| **Data source** | Clinical documents |
| **Standard** | 100% |

| **Title** | Participation of the centre in the register of living donors ^3^ |
| --- | --- |
| **Justification** | The data collected in the Living Donor Registry on the donor's state of health (before, immediately after and later in the post-donation period) provides LD transplant centres with absolutely necessary information for risk management, donation criteria and profiling in cases where living donation should be avoided due to the recipient's characteristics, even if there is a good and suitable donor (which is a new approach to living donation). |
| **Dimension** | Continuity of care |
| **Formula** | Number of actual living donors with follow-up data reported to the registry/ number of actual living donors |
| **Explanation of terms** | • Register: a national or supranational database where information is collected on all living donors.  • Minimum data reported for each living donor: as required by the national database system.  • The necessary data must be communicated to the registry within ÿÿ days of donation.  • Real living donor: an eligible living donor  • in whom an operative incision was made with the intention of recovering organs for the purpose of transplantation or from whom at least one organ was recovered for the purpose of transplantation. |
| **Population** | All real LDs who donated an organ during the period studied (for a period of ÿÿ days prior to data collection). |
| **Type** | Process |
| **Data source** | Consultation of the Living Donor Register |
| **Standard** | 100% |

| **Title** | Identifying potential living kidney donors ^3^ |
| --- | --- |
| **Justification** | Living donation is an important source of kidneys for transplantation in the context of organ shortages. From a medical point of view, the results of living donation Tx are better than those of cadaveric donation Tx, and donor nephrectomy is safe for the donor. There should be an efficient system for identifying potential living donors placed in hospitals with a transplant programme. |
| **Dimension** | Effectiveness |
| **Formula** | Number of potential living kidney donors assessed for donation by a specific group / number of potential kidney recipients x 100 |
| **Explanation of terms** | • Potential living donor: A legally competent person who has expressed their willingness to donate an organ while alive and is apparently suitable for living organ donation.  • Specific group: a multidisciplinary group independent of the recipient organisation, which aims to assess the donor (motivation, medical and socio-economic situation).  • Potential kidney recipient: patient on the renal waiting list. |
| **Population** | "New" patients on the waiting list in the period evaluated |
| **Type** | Results |
| **Data source** | The living donation team database. |
| **Standard** | 20% |

| **Title** | Long-term follow-up of living donors ^3^ |
| --- | --- |
| **Justification** | Evaluate the donor's post-donation situation and the implications of the donation process for LD. |
| **Dimension** | Security |
| **Formula** | Number of actual LDs with appropriate annual follow-up / number of actual LDs |
| **Explanation of terms** | • Real living donor: an eligible living donor• in whom an operative incision was made with the intention of recovering organs for the purpose of transplantation or from whom at least one organ was recovered for the purpose of transplantation Appropriate follow-up: at least the following ÿ requirements must be documented in the LD's medical records or alternative documentation: Medical condition, including relevant laboratory and imaging studies. Medical complications: wound infections, urinary tract infections, kidney stones, acute pyelonephritis and, very rarely (pneumothorax, pulmonary embolism). Psychosocial status (only in the first year). Post-donation regret of the decision to donate (satisfaction). Possible reduction in working capacity and other positive or negative consequences (socio-economic situation, etc.))  • If the donor is being followed up at another hospital, the name of the new hospital must be documented. In this case, the ÿ points above are unnecessary |
| **Population** | All living donors included in the register who were alive during the period studied |
| **Type** | Process |
| **Data source** | Medical records |
| **Standard** | 100% |

| **Title** | Evaluation of potential living donors ^3^ |
| --- | --- |
| **Justification** | The aim of donor evaluation is, on the one hand, to guarantee the viability and safety of the organ to be transplanted and, on the other, to ensure the long-term health of the donor. This indicator focuses on this second aspect, analysing the main issues related to donor health and donor evaluation. |
| **Dimension** | Security |
| **Formula** | All potential living donors who have been properly assessed / number of potential living donors who have been informed |
| **Explanation of terms** | Potential living donor: if you have been interviewed by a specialist (member of the donation team to become an organ donor). |
| **Population** | All potential TAs who were informed about the donation during the period studied. |
| **Type** | Results |
| **Data source** | Medical records and living donor registry or other alternative records for LD information during the period studied. |
| **Standard** | 80% |

| **Title** | Percentage of kidney transplants from living donors ^1^ |
| --- | --- |
| **Formula** | Number of kidney tests carried out on living donors / total no. of tests carried out x 100 |
| **Type** | Results |
| **Standard** | 10% |

| **Title** | Laparoscopic living donor nephrectomies ^1^ |
| --- | --- |
| **Formula** | No. of laparoscopic nephrectomies on living donors performed / total no. of nephrectomies on living donors x 100 |
| **Type** | Results |
| **Standard** | 60% |

| **Title** | Percentage of individuals registered as potential living kidney donors who are considered fit to donate ^11^ |
| --- | --- |
| **Dimension** | Equitable |
| **Formula** | **Denominator: total number of individuals registered as potential living donorsNumerator: number of people in the denominator who are considered able to donate** |
| **Explicação dos termos** | **Points to consider for implementation:**  (i) consider the percentage of people deemed fit to donate after passing the initial medical and social questionnaire;  (ii) do not include potential donors who withdraw from the process;  (iii) stratify by demographic and risk factor categories (e.g. race, female donors, donors >60 years, number with treated hypertension, etc.) |

| **Title** | Percentage of living kidney donors who develop end-stage renal disease ^11^ |
| --- | --- |
| **Dimension** | Security |
| **Formula** | **Denominator: total number of living kidney donors** **Numerator: number of people in the denominator who develop end-stage renal disease, defined as needing dialysis, receiving a kidney transplant or receiving conservative care** |

| **Title** | Percentage of living kidney donors who die during the initial hospitalisation for donation ^11^ |
| --- | --- |
| **Dimension** | Security |
| **Formula** | **Denominator: total number of living kidney donors** **Numerator: number of people in the denominator who die during the initial hospitalisation for donation** |

| **Title** | Percentage of living kidney donors who suffer a serious safety event during the initial hospitalisation period for donation ^11^ |
| --- | --- |
| **Dimension** | Security |
| **Formula** | **Denominator: total number of living kidney donors** **Numerator: number of people in the denominator with a serious safety event during the initial period of hospitalisation for donation** |
| **Explanation of terms** | **Note: a serious safety event can be an erroneous and/or incompatible blood transfusion, major medication error, retained foreign body, pressure ulcer, fracture, fall, etc.** |

| **Title** | Percentage of living kidney donors who had a complication in the first 30 days after donation surgery ^11^ |
| --- | --- |
| **Dimension** | Efficacy |
| **Formula** | **Denominator: total number of living kidney donors** **Numerator: number of people in the denominator who had a complication in the first 30 days after the donation surgery** |
| **Explanation of terms** | DVT (deep vein thrombosis) / PE (pulmonary embolism)  **Note: a complication can be an infection (pneumonia, urinary tract infection, bacteraemia, surgical site infection); cardiovascular (myocardial infarction, cardiac arrest, DVT, PE, stroke); blood transfusion; or the need for an unplanned operation.**  **Points to consider for implementation: overall measure of the 30-day complication rate, as well as separate measures for infection, cardiovascular, transfusion and unplanned operation** |

| **Title** | Percentage of living kidney donors with unplanned readmission to any hospital within 30 days of discharge after donation surgery ^11^ |
| --- | --- |
| **Dimension** | Efficacy |
| **Formula** | **Denominator: total number of living kidney donors discharged from hospita within 30 days**  **Numerator: number of people in the denominator who have an unplanned readmission to any hospital within 30 days of discharge after donation surgery** |

| **Title** | Percentage of living kidney donors with a long-term follow-up plan ^11^ |
| --- | --- |
| **Dimension** | Efficacy |
| **Formula** | **Denominator: total number of living kidney donors discharged from hospitalNumerator: number of people in the denominator who have a long-term follow-up plan**  **Points to consider for implementation:** (i) measured at the time of the early postoperative follow-up visit; (ii) follow-up can be with a primary care provider or a living kidney donor programme, as long as the plan is documented |

| **Title** | Percentage of potential living kidney donors who report a high level of satisfaction with the educational resources provided ^11^ |
| --- | --- |
| **Dimension** | Patient-centred |
| **Formula** | **Denominator: total number of individuals registered as potential living donors** **Numerator: number of people in the denominator who report a high level of satisfaction with the educational resources provided**  **Note: educational resources (e.g. information sessions, videos, handouts) provided by the living kidney donor team**.  **Points to consider for implementation:**  (i) questionnaire will need to be developed;  (ii) consider separate measures for those who have donated versus those who have not |

| **Title** | Percentage of potential living kidney donors who report a high level of satisfaction with the care received during the pre-donation assessment process ^11^ |
| --- | --- |
| **Dimension** | Patient-centred |
| **Formula** | **Denominator: total number of individuals registered as potential living donors** **Numerator: number of people in the denominator who report a high level of satisfaction with the care received**  **Points to consider for implementation:**  (i) questionnaire will need to be developed; (ii) consider separate measures for those who have donated versus those who have not” |

| **Title** | Percentage of living kidney donors who report a high level of satisfaction with the care received during the donation hospitalisation ^11^ |
| --- | --- |
| **Dimension** | Patient-centred |
| **Formula** | **Denominator: total number of living kidney donors** **Numerator: number of people in the denominator who report a high level of satisfaction with the care received during hospitalisation**  **Points to consider for implementation: questionnaire will need to be developed** |

| **Title** | Percentage of living kidney donors who report a high level of satisfaction with the care received in the post-donation clinic ^11^ |
| --- | --- |
| **Dimension** | Patient-centred |
| **Formula** | **Denominator: total number of living kidney donors** **Numerator: number of people in the denominator who report a high level of satisfaction with the care received at the post-donation clinic.**  **Points to consider for implementation: questionnaire will need to be developed** |

| **Title** | Number of days from when the person registers as a potential living kidney donor to when suitability is determined ^11^ |
| --- | --- |
| **Dimension** | Opportunity |
| **Formula** | **Calculation: can be measured as the average, median or distribution of waiting times (in days) from when the individual registers as a potential living donor to when suitability is determined**  **Points to consider for implementation: consider measuring the percentage that completes the evaluation within the defined times (e.g. 3 months, 6 months)** |

| **Title** | Number of days from the time the person is considered a suitable living kidney donor for surgery ^11^ |
| --- | --- |
| **Dimension** | Opportunity |
| **Fornula** | **Calculation: can be measured as the average, median or distribution of waiting times (in days) from when a person is considered a living donor fit for donation surgery.** |

| **Title** | Number of days between admission and discharge after living kidney donation (length of stay) ^11^ |
| --- | --- |
| **Dimension** | Efficient |
| **Formula** | **Calculation: can be measured as the mean, median or distribution of hospitalisation time (in days) from the patient's hospitalisation time until discharge after living kidney donation.** |

**Referências**

1. Cienfuegos - Belmonte IR, León Dueñas E, Román - Martín AA, Olmo-Ruíz M, González- Roncero FM, Medina- López RA. Evaluation of the Spanish Urogical Assoation quality care incators in a kidney transplantation programme. Actas Urologicas Espanolas [Internet]. 2016 Oct 1;40(8): 523-8. Available from: <https://pubmed.ncbi.nlm.nih.gov/26992850>
2. ‌Elcock - Straker B, Manyalich Vidal M, Gomez MP. Kidney Donation and Transplant Outcomes in Trindad and Tobago: A 15-Year Experience of the National Organ Transplant Unit. Experimental and Clinical Transplantation. 2022 Jul;20(7):649-56
3. Guide to the quality and safety of organs for transplantation - European Directorate for the Quality of Medicines & HealthCare - EDQM [Internet]. European Directorate for the Quality of Medicines & HealthCare. 2022. Available from: <https://www.edqm.eu/en/guide-quality-and-safety-of-organs-for-transplantation>
4. Glavinovic T, Vinson AJ, Silver SA, Yohanna S. An Environmental Scan and Evaluation of Quality Indicators Across Canadian Kidney Transplant Centers. Canadian Journal of Kidney Health and Disease. 2021 Jan;8:205435812110279.
5. Schaffhausen CR, Bruin MJ, Chu S, Wey A, Snyder JJ, Kasiske BL, et al. The importance of transplant program measures: Surveys of three national patient advocacy groups. Clinical Transplantation. 2018 Oct 31;32(12):e13426.
6. Toussaint ND, McMahon LP, Dowling G, Soding J, Safe M, Knight R, et al. Implementation of renal key performance indicators: Promoting improved clinical practice. Nephrology. 2015 Feb 24;20(3):184–93.
7. Treviso P, Brandão FH, Saitovitch D. Identificação de indicadores de qualidade para um serviço de transplante renal. Brazilian Journal of Transplantation. 2010 Jun 1;13(3):1345–8.
8. Organ Donation European Quality System Q ✓ ODEQUS Quality Criteria & Quality Indicators in Organ Donation [Internet]. [cited 2024 Jul 2]. Available from: <http://www.odequs.eu/pdf/ODEQUS_Quality_Criteria-Indicators.pdf>
9. Brett KE, Ertel E, Grimshaw J, Knoll GA. Perspectives on Quality of Care in Kidney Transplantation. Transplantation Direct. 2018 Sep;4(9):e383.
10. Habbous S, Barnieh L, Litchfield K, McKenzie S, Reich M, Lam NN, et al. A RAND-Modified Delphi on Key Indicators to Measure the Efficiency of Living Kidney Donor Candidate Evaluations. Clinical journal of the American Society of Nephrology: CJASN [Internet]. 2020 Oct 7 [cited 2024 Jul 2];15(10):1464–73. Available from: <https://pubmed.ncbi.nlm.nih.gov/32972951/>
11. Knoll GA, Fortin MC, Gill J, Grimshaw JM, Hartell DP, Karnabi P, et al. Measuring quality in living donation and kidney transplantation: moving beyond survival metrics. Kidney International. 2020 Oct;98(4):860–
12. Brett KE, Ritchie LJ, Ertel E, Bennett A, Knoll GA. Quality Metrics in Solid Organ Transplantation. Transplantation. 2018 Jul;102(7):e308–30.
13. Graham JM, Courtney AE. The Adoption of a One-Day Donor Assessment Model in a Living Kidney Donor Transplant Program: A Quality Improvement Project. American Journal of Kidney Diseases. 2018 Feb;71(2):209–15.
14. Hermanowicz M, Borczon S, Lewandowska D, Przygoda J, Podobińska I, Danielewicz R, et al. Quality System of Kidney Donation for Transplantation From Living Donors in Poland. Transplantation Proceedings. 2020 Sep;52(7):2033–5.
